# Case-based medical informatics

**DOI:** 10.1186/1472-6947-4-19

**Published:** 2004-11-08

**Authors:** Stefan V Pantazi, José F Arocha, Jochen R Moehr

**Affiliations:** 1 School of Health Information Science, University of Victoria, BC, Canada; 2 Department of Health Studies and Gerontology, University of Waterloo, Ont., Canada

## Abstract

**Background:**

The "applied" nature distinguishes applied sciences from theoretical sciences. To emphasize this distinction, we begin with a general, meta-level overview of the scientific endeavor. We introduce the *knowledge spectrum *and four interconnected modalities of knowledge. In addition to the traditional differentiation between implicit and explicit knowledge we outline the concepts of general and *individual knowledge*. We connect general knowledge with the "frame problem," a fundamental issue of artificial intelligence, and individual knowledge with another important paradigm of artificial intelligence, *case-based reasoning*, a method of individual knowledge processing that aims at solving new problems based on the solutions to similar past problems.

We outline the fundamental differences between Medical Informatics and theoretical sciences and propose that Medical Informatics research should advance individual knowledge processing (case-based reasoning) and that natural language processing research is an important step towards this goal that may have ethical implications for patient-centered health medicine.

**Discussion:**

We focus on fundamental aspects of decision-making, which connect human expertise with individual knowledge processing. We continue with a knowledge spectrum perspective on biomedical knowledge and conclude that case-based reasoning is the paradigm that can advance towards personalized healthcare and that can enable the education of patients and providers.

We center the discussion on formal methods of knowledge representation around the frame problem. We propose a context-dependent view on the notion of "meaning" and advocate the need for case-based reasoning research and natural language processing. In the context of memory based knowledge processing, pattern recognition, comparison and analogy-making, we conclude that while humans seem to naturally support the case-based reasoning paradigm (memory of past experiences of problem-solving and powerful case matching mechanisms), technical solutions are challenging.

Finally, we discuss the major challenges for a technical solution: case record comprehensiveness, organization of information on similarity principles, development of pattern recognition and solving ethical issues.

**Summary:**

Medical Informatics is an applied science that should be committed to advancing patient-centered medicine through individual knowledge processing. Case-based reasoning is the technical solution that enables a continuous individual knowledge processing and could be applied providing that challenges and ethical issues arising are addressed appropriately.

## Background

### A meta-level view of science

Our aim is to place Medical Informatics in the context of other sciences and to bring coherence in its formal education [1]. This will necessarily place the discussion at a meta-level view of science, which traditionally was the concern of philosophers. From such a general perspective, science could be defined as "the business of eliciting *theories *from observations in a certain *context*, with the hope that those theories will help to understand, predict and solve problems." Also revolving around the "business of creating theories," R. Solomonoff's ideas [2], summarized in [3], contribute to the basis of Algorithmic Information Theory (AIT) [4], a relatively new area of research initiated by A. Kolmogorov, R. Solomonoff and G. Chaitin, and regarded as the unification of Computer Science and Information Theory. According to Solomonoff's view, a scientist's theories are compressions of her observations (i.e., her experimental data). These compressions are used to explain, communicate and manage observations efficiently and, if valid, to help solving problems, understanding and predicting. Intuitively, the higher the compression achieved by the theory, the more "elegant" that theory and the higher its chances of acceptance. This very general perspective of the scientific endeavor also makes science to appear twofold: it comprises the creation of theories (i.e., theory elicitation) as well as their subsequent use in understanding, predicting and solving problems (i.e., theory application). Therefore, science seems to be driven by two opposite forces: that of creating theories, and that of applying those theories to practical applications.

The four-dimensional space-time continuum we live in (i.e., our universe) forms the reality (i.e., the context) of all scientific observations. The compression of the immense complexity and dynamicity of this reality in concise "theories of everything" was already demonstrated by Zuse [5,6] and recently Schmidhuber [7]. These results of theoretical computer science demonstrate the power of human theory elicitation and provide important answers to old questions of science and philosophy. However, their unfeasibility when applied to practical problems, which would be equal to building computing devices capable of running precise simulations of our reality, also widens the gap between theoretical research and practical sciences. For the time being, humanity still needs to divide science and define *human knowledge *as a collection of individual theories elicited from scientific observations. The immense number of theories that comprise the collective human knowledge about every possible subject, as well as its extraordinary dynamics, have forced us to divide it into what we commonly refer to as *knowledge domains*, thereby reducing the contexts of our observations to smaller space-time continuums. The attempts to process with computers the knowledge in a domain have taught us that we need to recognize the reality of the "knowledge acquisition bottleneck" [8] and to not underestimate the importance of common-sense knowledge (see [9] and [10-13]). The particularities with regard to the context retention, acquisition, representation, transferability and applicability of domain knowledge, causes us to distinguish between different modalities of domain knowledge, and place them on what we refer to as the *knowledge spectrum*.

### The knowledge spectrum

**Figure 1:**
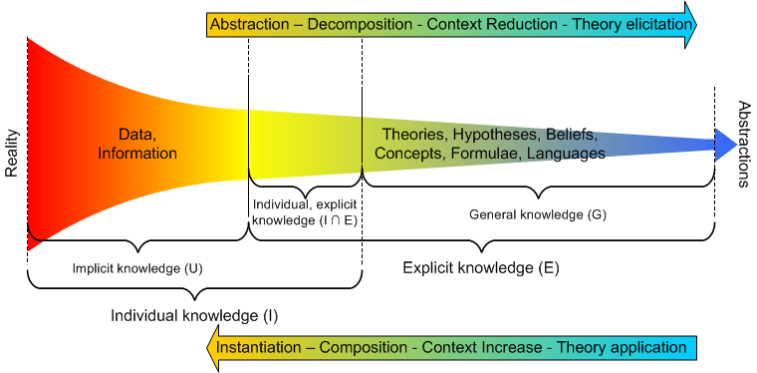
The knowledge spectrum.

The knowledge spectrum (Figure 1) spans from a *complex reality *(the source of experimental data and information gathered from observations and measurements) to *high-level abstractions *(e.g., theories, hypotheses, beliefs, concepts, formulae etc). Therefore, it comprises increasingly lean modalities of knowledge and knowledge representations media and the relative boundaries and relationships between them. Two forces manifest on the knowledge spectrum: that of creating abstractions and that of instantiating abstractions for practical applications. The former is the theory elicitation and is synonymous to processes of context reduction and knowledge decomposition. The latter, theory application, equates to context increase and knowledge composition processes. The engines behind the two knowledge spectrum forces are the knowledge processors, natural or artificial entities able to create abstractions from data and to instantiate abstractions in order to fit reality.

Knowledge is traditionally categorized into *implicit *and *explicit *(Table 1) and ranges from rich representations grounded in a reality, to highly abstracted, symbolic representations of that reality. The classical distinction between data, meta-data, information, knowledge and meta-knowledge is simplified by our subscription to the unified view of Algorithmic Information Theory (AIT) [4] which recasts all knowledge modalities and their processing into a general framework requiring a Universal Turing Machine, its programs and data represented as finite binary sequences. From this perspective a precise distinction between these modalities becomes unimportant.

*Implicit knowledge *(U, from unobvious, unapparent) is the rich, experiential, sensorial kind of knowledge that a *knowledge processor *acquires when immersed into an environment (i.e., grounded in an environment), or presented with detailed representations of that environment (e.g., images, models, recordings, simulations). It is very well applicable to specific instances of problems and relies on processing mechanisms such as feature selection, pattern recognition and associative memory.

*Explicit knowledge *(E) is the abstract, symbolic type of knowledge present explicitly in documentations of knowledge such as textbooks or guidelines. It requires a representation language and the capability of a knowledge processor to *construe the meaning of concepts *of that language. It is applicable to both specific and generic problems and relies on explicit reasoning mechanisms.

The distinction between implicit and explicit knowledge are useful to characterize the nature of human expertise, but become problematic when one wants to describe fundamental differences between theoretical and applied sciences: many applied sciences, especially knowledge intensive ones, in addition to general theories of problem solving, also make use of *explicit knowledge *in order to describe, with various degrees of precision, particular instances of problem solving and theory application. This represents the rationale for further dividing the knowledge spectrum into *general and individual knowledge *(Table 2).

### General knowledge

*General knowledge *(G) is the explicit, abstract, propositional type of knowledge (e.g., guidelines), well applicable to context-independent, generic problems. However, it is more difficult to use in specific contexts because of the gap between the general knowledge itself and a particular application context. This knowledge gap translates into uncertainty when a general knowledge fact is instantiated to a specific situation. For example, knowing generally that a certain drug may give allergic reactions but being uncertain whether a particular patient may or may not develop any, is an example of what we consider the uncertainty associated with general knowledge. The creation of general knowledge (i.e., abstraction, generalization, context reduction, theory elicitation) is a relevance-driven process done by "stripping away irrelevancies" [9]. This causes general knowledge to have a lower complexity and be more manageable: "generalization is saying less and less about more and more" [9].

**Figure 2:**
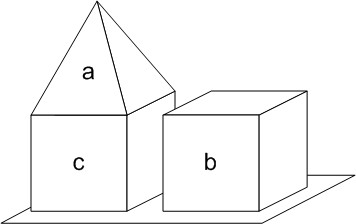
**A blocks world example. **In this particular example expressions such as: on(a, c), on(c, table), on(b, table), pyramid(a), brick(b), brick(c), ¬same-as(a, c), same-as(b, c), etc., are true.

Formal representations of explicit knowledge have been common in early artificial intelligence (AI) applications in the context of expert system development. They operated under the "closed world assumption" and were meant to make the representation of knowledge manageable, reproducible and clear. However this assumption also rendered the expert systems "brittle" or completely unusable when applied to real world problems [14]. The completeness necessary for automatic reasoning using explicit reasoning mechanisms can be illustrated with the following formal definition of the concept of "a brick" in a limited, hypothetical world, containing only simple geometric objects such as bricks and pyramids (Figure 2) (adapted from [15]): "being a brick implies three things:

   1. first, that the brick is on something that is not a pyramid;

   2. second, that there is nothing that the brick is on and that is on the brick as well; and

   3. third, that there is nothing that is not a brick and the same thing as the brick."

This definition could have the predicate calculus representation:


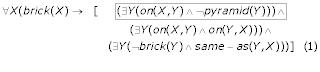


This representation shows that an intelligent agent who has no implicit knowledge of the hypothetical physical world and no capacity of generalization or analogy making, must be explicitly provided with all knowledge necessary to reason about "bricks" in that limited reality. Such approaches are known to suffer from a fundamental shortcoming, the "frame problem."

### The frame problem

Daniel Dennett was the first philosopher of science who clearly articulated the "frame problem" and promoted it as one of the central problems of artificial intelligence [16] (also see [17]). Janlert [18] identifies the frame problem with "the problem of representing change." In [14] the frame problem is defined as "the problem of representing and reasoning about the side effects and implicit changes in a world description." In order to articulate and circumvent the abstract nature of its definition, Dennett has invented a little story involving three generations of increasingly sophisticated robots. These fictitious robots are products of early artificial intelligence (AI) technology that use automated reasoning based on formal representations similar to the brick example. These particular robots are specifically designed to solve a problem consisting of the retrieval of their life-essential batteries from a room, under the threat of a ticking bomb set to go off soon. Although increasingly sophisticated in their reasoning, all three successive versions of the robot fail:

• The first robot fails by *missing a highly relevant side effect *of pulling the wagon with the batteries out of the room: the ticking bomb sitting on the same wagon was also retrieved, together with the batteries.

• The second robot *did not finish its extensive, irrelevant side-effect reasoning procedures *before the bomb goes off. As Dennett ironically puts it, the robot "had just finished deducing that pulling the wagon out of the room would not change the color of the room's walls and was embarking on a proof of the further implication that pulling the wagon out would cause its wheels to turn more revolutions than there were wheels on the wagon – when the bomb exploded."

• The third robot failed because it was "*busily (i.e., explicitly) ignoring some thousands of implications it has determined to be irrelevant*" and its batteries were therefore lost in the inevitable explosion.

*The frame problem can therefore be recast as a problem of relevance *[17]*(see preface), which is compounded by time constraints. It demonstrates that relevance judgment mechanisms based on general knowledge are time consuming and cause the failure to solve time-constrained decision problems. It is a problem **only because in the real world we do have time constraints***.

### Individual knowledge

*Individual knowledge *(I) or instance specific knowledge, on the other hand, is a knowledge modality very well applicable to real problems, because it *identifies uniquely *and matches precisely an application context. The knowledge gap and uncertainty are reduced but still exist because of our changing reality (time dimension) which may render individual knowledge about a patient collected in the past (e.g., value of blood pressure from a month ago), less applicable in the present or future. Because it preserves context (i.e., it is more grounded), individual knowledge has a higher complexity than general knowledge and hence is more difficult to manage (i.e., has high memory requirements). For example, knowing the drugs and the precise description (e.g., numeric, textual, visual) of the allergic reactions that they caused in a certain person, as well as many other particular knowledge facts about individual, is what we call individual knowledge. The uncertainty and knowledge gap related to the application of such knowledge to future instances of decision making involving that individual are reduced: individual knowledge is supposed to fit very well the application context where it was originally captured.

### Case-based reasoning

Individual knowledge captured from a very specific context (e.g., diagnosing a particular patient with a particular disease) can be extrapolated to similar contexts. The higher the similarity between contexts, the smaller the knowledge gap and instantiation uncertainty and the higher the chances for a successful solution to a new problem. For this reason, individual knowledge processing has become increasingly important for artificial intelligence applications and is defined as the approach to solving new problems based on the solutions of similar past problems [14,19-21]. It has several flavors (e.g., exemplar-based, instance-based, memory-based, analogy-based) [21] which we will refer to in this paper interchangeably, through the generic term of "case-based reasoning" (CBR).

There are four steps (the four "RE") that a case-based reasoner must perform [14,20,21]:

1. RETRIEVE: the retrieval from memory of the cases which are appropriate for the problem at hand; this task involves processes of analogy-making or case pattern matching;

2. REUSE: the decomposition of the retrieved cases in order to make them applicable to the problem at hand;

3. REVISE: the compositional adaptation and application of the knowledge encoded in the retrieved cases to the new problem; and

4. RETAIN: the addition of the current problem together with its resolution to the case base, for future use.

CBR entails that an expert system has a rich collection of past problem-solving cases stored together with their resolutions. CBR also hinges on a proper management of the case base and on appropriate mechanisms for the matching, retrieval and adaptation of the knowledge stored in the cases relevant to a new problem. Ideally, the individual knowledge in a case-base will progress asymptotically towards an exhaustive knowledge base, which represents the "holy grail" of knowledge engineers. From a learning systems point of view, similarly to artificial neural networks [22,23] and inductive inference systems [24] that learn from training examples, a CBR system acquires new knowledge, stores it in a case base and makes use of it in new problem solving situations.

**Figure 3:**
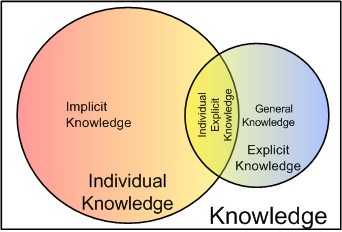
The relationships between the knowledge modalities.

The absolute positions and shapes of boundaries between the four knowledge modalities, although admittedly not as precise as drawn on the knowledge spectrum in Figure 1, are not of importance for this discussion. However, the relative relationships between knowledge modalities are, and can be represented formally as a Venn diagram (Figure 3), which implies that:

• Individual knowledge has a higher complexity than the explicit knowledge elicited from the same context. This is equivalent to stating that, for example, the picture of a person encodes more knowledge than the textual description of that person's appearance.

• Implicit knowledge is a subset of the individual knowledge.

• General knowledge is a subset of the explicit knowledge.

• The set of individual knowledge represented explicitly formed by the intersection of individual knowledge with explicit knowledge is a nonempty set. This is equivalent to stating that it is possible, for example, for an explicit textual description to identify a context uniquely (e.g., the complete name and address of a person at a specified moment in time).

### A meta-level view of Medical Informatics

The meta-level overview of sciences and the definitions and properties of the knowledge spectrum and knowledge modalities enable us to draw some fundamental differences between theoretical sciences and applied sciences such as Medicine [25] and Medical Informatics. From this perspective, theoretical sciences (e.g., theoretical computer science):

• Make use of observations which are highly abstract symbolisms and create far more limited contexts of application of their theories, when compared to the complexity of the human body or of any social or biological system,

• Have as a primary purpose the creation of *general knowledge *comprising valid, powerful theories which explain precisely and completely the observations, and therefore,

• Include a relatively limited number of precise theories which are evaluated primarily by their power of explaining experimental observations, elegance, generality, and

• Are less concerned with the acquisition of the *individual knowledge *required by the practical implementation and by the application of results to real world problems.

Applied sciences such as Medicine and Medical Informatics, on the other hand:

• Gather extensively data and observations (*individual knowledge*) from very complex systems [9,26] (e.g., human body), which are characterized by high individual variation and randomness;

• Have as a primary purpose not only distilling data and observations into *general knowledge*, but are also concerned with the implementation details and with the application of theories to individual problem solving (e.g., diagnosis and treatment of real patients),

• May lack the incentive to refine existing theories which are objectively wrong as long as practical success is achieved [25],

• Contain very few simple, "elegant" theories (*general knowledge*) that can solve individual problems completely or explain and predict accurately [27] because of the complexity of the human body and its individual variation and, therefore,

• May pursue the application of a multitude of mutually contradictory, poorly grounded, general theories (e.g., the general theory of medical reasoning and the concepts of "diagnosis" and "symptom") [1,25],

• Abound in general theories (e.g., guidelines) which are "lossy" (i.e., ignore individual context variation) and which are evaluated statistically by their practical success relative to existing ones (e.g., cancer therapy),

• Attempt to make up for the *knowledge gap *between *general knowledge *and the *reality *where knowledge is applied, by employing experienced clinicians who require extensive training and information technology (e.g., decision support), and, in addition,

• Are compounded by time-constrained circumstances and largely unsolved ethical issues (e.g., privacy and confidentiality, genomics research).

Given the special circumstances of our applied science in the context of other sciences and the increasing recognition of the importance of knowledge processing to Medical Informatics [28], we propose, as part of the thesis of this paper, that *Medical Informatics should complement the traditional quest for general biomedical knowledge with the advance of acquisition, storage, communication and use of **individual knowledge**. By doing so, Medical Informatics will provide a solution to the problems that arise during the use of general knowledge and, in the same time, will enable clinical research as well as advanced decision support and education of both healthcare providers and patients*.

Individual knowledge processing equates to a case-based reasoning (CBR) approach that employs collections of patient cases. Currently, such collections are the focus of research on Electronic Health Records (EHR). Envisioned as "womb to tomb" collections of patient-specific data, EHR contain a wealth of data that could be used to support case-based decisions. *If EHR are to be used in a CBR context, the issues pertinent to the design of case-bases automatically become pertinent to the EHR design, and the CBR paradigm becomes important to Medical Informatics*.

**Figure 4:**
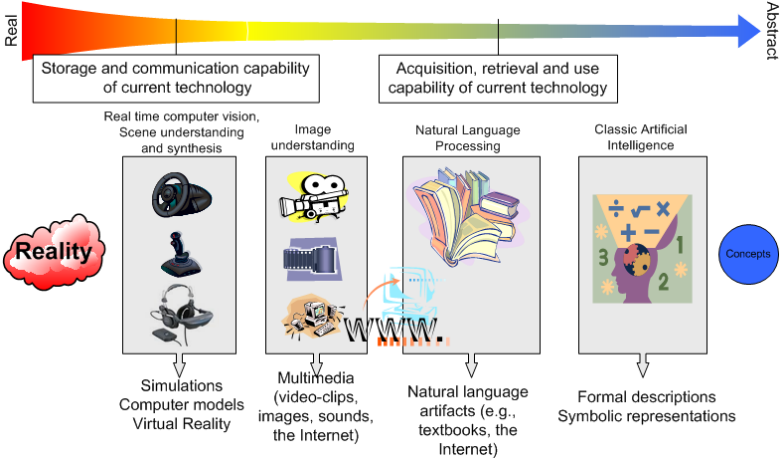
**Knowledge representation media on the knowledge spectrum. **The storage and transmission of knowledge are more advanced compared to the knowledge acquisition, retrieval and use capability of current technology.

The overall *knowledge processing capacity *of healthcare systems can be thought to be distributed between two sources: human resources (i.e., healthcare professionals) and information technology (Medical Informatics). An ideal CBR approach would increase this knowledge processing capacity by allowing for the automatic processing (acquisition, representation, storage, retrieval and use) of individual knowledge present in increasingly rich knowledge media such as natural language artifacts, images, videos and computer simulations of reality (Figure 4). The storage and communication of knowledge are well advanced by current information technology. However, most of the acquisition, retrieval and knowledge use are, and will continue to be the task of professionals until advanced processing (e.g., real-time computer vision, scene understanding and synthesis, image understanding, robotics, natural language understanding) are applicable. *Given the widespread use of natural languages as knowledge representation and communication media, it follows that natural language processing (NLP) research is a very important component of Medical Informatics, required to advance the organization and processing of individual knowledge in reusable case-bases. Further, the goal to advance processing of increasingly complex knowledge representations (e.g., natural language, sounds, images, simulations) and create intelligent machines that can hear, see, think, adapt and make decisions, brings Informatics even closer to what traditionally was the concern of Artificial Intelligence (AI)*.

Finally, because the knowledge processing capacity of human resources tends to remain relatively constant, *moving towards the ideal of individual knowledge processing, no matter how slowly, may also have ethical implications because it proves that medical informaticians are trying to do everything they can in order to serve the interest of the individual*.

## Discussion

In order to support our thesis, the following discussion will focus initially on fundamental aspects of medical decision-making and biomedical knowledge creation from the standpoint of the knowledge spectrum. This will lead to a discussion of fundamental knowledge representation and processing principles and the proposal of a CBR perspective on EHR, including challenges and potential solutions.

### Human and computer knowledge processing

#### Decision making in medicine

Medicine is a knowledge intensive domain where time-constrained decisions based on uncertain observations are commonplace. In order to successfully cope with such situations, health professionals go through a tedious learning process in which they gain the necessary domain knowledge to evolve from novices to experts. As experts, health professionals have attained, among other things, two important, highly interrelated abilities:

• To be able to reduce knowledge complexity by *determining efficiently what is relevant *for solving a problem in a particular situation, and,

• To be able to *reduce the knowledge gap *between knowledge facts and reality which translates into being able to reduce the uncertainty of knowledge instantiation to a particular context.

For example, both the presence and the absence of a past appendectomy are relevant and contribute (potentially unequally) to reducing the uncertainty of instantiation of the biomedical knowledge of an expert to a particular context of a patient with right lower abdominal pain. Fundamental to decision making, relevance judgments and uncertainty reduction seem both closely connected with the quality and quantity of knowledge available for solving a problem as well as with the nature of knowledge processing mechanisms. Studies of expert-novice differences in medicine [29] have shown that the key difference between novices and experts is the highly organized knowledge structures of the latter, and not the explicit strategies or algorithms they use to solve a problem. This is supported by expert system development experiences which showed that a system's power lies in the domain knowledge rather than in the sophistication of the reasoning strategies [14]. Studies of predictive measures of students' performance indicate that tests which measure the acquisition of domain knowledge are the best predictors [30]. The work on naturalistic decision-making (NDM) and the development of psychological models of "recognitional decision-making" such as the Recognition-Primed Decision (RPD) [31-33], suggest the heavy dependence of decision makers on their previous experience of problem-solving and also on their ability to perform mental simulations.

The discussion around the amount of problem solving experience of a decision maker becomes critical in time-constrained decision circumstances. The exhaustiveness of the knowledge base and the efficiency of retrieval mechanisms now become paramount to the decision speed. Empirical evidence that shows the existence of "systematic changes of cognitive processes" related to time stress, comes from the studies on the psychology of decision-making under time constraints [34]. Although most of these studies attest the overall negative effect of time stress on the "effectiveness of decision-making processes" [35], others [31,33] argue that even extremely time-constrained situations could be handled successfully by human subjects, given enough expertise (i.e., enough problem solving experience).

Since humans are able to make sound relevance judgments and reduce instantiation uncertainty of knowledge most of the times, the following questions arise: What is their strategy for increasing the exhaustiveness of their knowledge base while managing its exponential complexity? How do they represent and organize their knowledge and how do they manage time-constrained situations? At least some of these questions have been under intense scrutiny that has resulted in important empirical work on naturalistic decision-making [32,33,36,37]. Important insights have been gained at the individual but also at the organizational and social levels. Coherent with the importance of the social aspects of decision-making, Armstrong [38] builds an interesting argument about the Darwinian evolution, social networking and the drive for knowledge discovery of the humanity as being some of the reasons that contribute to the human decision making potential.

**Figure 5:**
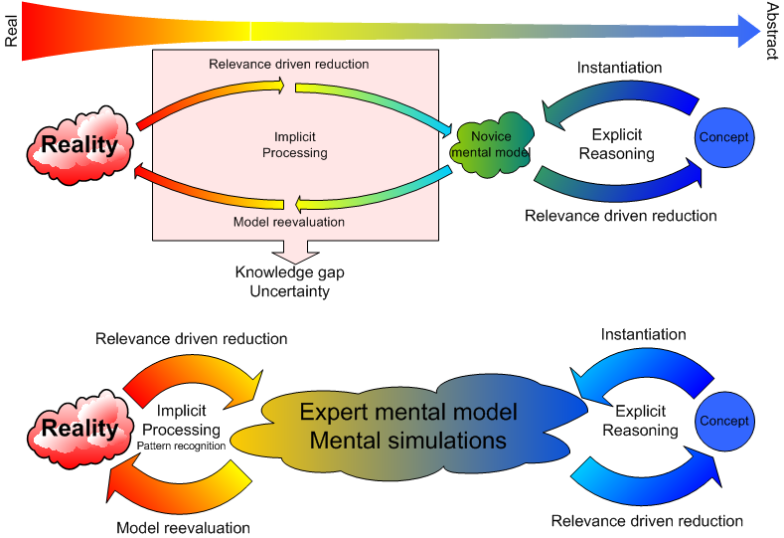
Knowledge representation and processing in novices and experts.

From the perspective of the knowledge spectrum, it seems reasonable to associate expert decision makers with individual knowledge and novices with the more abstract general knowledge about a subject, available in explicit knowledge artifacts (e.g., textbooks, guidelines). It is also conceivable that mental models of experts span a great length of the knowledge spectrum, causing them to efficiently perform implicit processing (feature selection, pattern recognition, associative recall) and also just-in-time explicit reasoning (Figure 5). The ability to move freely across the knowledge spectrum causes experts to efficiently reduce data to abstractions and to create hypotheses and micro-theories through sound relevance judgments. The powerful mental simulations that experts can perform allow them to construe appropriate meanings of concepts and to verify their hypotheses against contexts of reality.

Novices, on the other hand, have limited mental models of reality situated towards the abstract region of the spectrum. This causes them to have difficulties with construing appropriate meanings of concepts due to the increased knowledge gaps between their mental models and reality. Novices are therefore unable to make sound relevance judgments and limited in their ability of interpreting data and creating abstractions. They are also usually overwhelmed by the explicit, general knowledge present in textbooks and guidelines and unable to fully construe the meanings of concepts present in such knowledge artifacts.

*In conclusion, in information and knowledge intensive domains such as medicine, explicit reasoning is important but individual knowledge acquisition (i.e., experience) and processing (i.e., CBR) are crucial for decision-making. Because the nature of expertise seems largely connected with individual knowledge processing, it follows that the evolution of novices into experts is unattainable only by the provision of extensive general knowledge. In addition, not only the individual learning but also the collective sharing of experiences (e.g., case records, personal stories, etc.) between individuals and between generations, contribute to the way humans deal with decision problems*.

### Patient-centered vs. population-centered healthcare

The major driving force of science is universally applicable knowledge (i.e., general knowledge). While creating and communicating new knowledge, scientists move across the knowledge spectrum from the data that captures the reality of their experiments and observations towards abstract representations that allow them to communicate their theories. In biomedical research, such an example is the randomized controlled trial (RCT), currently regarded as the gold standard for knowledge creation. The correct design of an RCT is crucial for the validity of the medical evidence obtained. A correct randomization process in RCTs will limit the bias and increase the chance for applicability of the evidence obtained, to a specifically selected group of patients (e.g., "women aged 40–49 without family history of breast cancer"). However, at the same time, the randomization process removes the circumstances of individual cases and creates a knowledge gap between the RCT evidence and future application instances. As with any statistical approach, the RCT-based evidence is best applicable at the population level rather than at the individual level.

This depersonalization of medical knowledge and evidence was also noted by others [39,40] and could also be illustrated by the observation that most patients feel relieved when told that the chances of being successfully treated for a certain condition are 99%, for example. Although this is psychologically very positive, the patients should not necessarily be relieved, as they could very well happen to fall among the 1%, for whom things could go wrong and for whom, usually, the RCT-based evidence does not provide additional information. An experienced physician and, from a CBR perspective, a highly efficient case-based reasoner, is most of the times able to individualize the medical decision for a particular patient for whom things are likely to go wrong and fill in the knowledge gap between the RCT evidence and the medical problem at hand. This could lead to avoiding a therapeutic procedure recommended by the medical evidence. The individual knowledge that this decision is based on is usually not provided by the RCT, but is acquired through a tedious process of training. This decision is often so complex that it cannot be easily explained as it becomes heuristic in nature and is motivated by the individual knowledge that a decision maker possesses.

Others [41] have also pointed out that when physicians manage their cases (e.g., diagnosis and treatment), their previous experience allows them to make informed decisions based on heuristics rather than on a sound, complete and reproducible reasoning, such as logical inference based on a predicate calculus representation of a problem. In addition, human experts often disregard probabilistic, RCT-type of evidence and consistently detach themselves from the normative models of classical decision theory (e.g. probability theory, Bayes theory) in favor of heuristics-based approaches. Although prone to occasional failures, heuristics-based decisions are much more efficient in time-constrained and uncertain situations [33].

**Figure 6:**
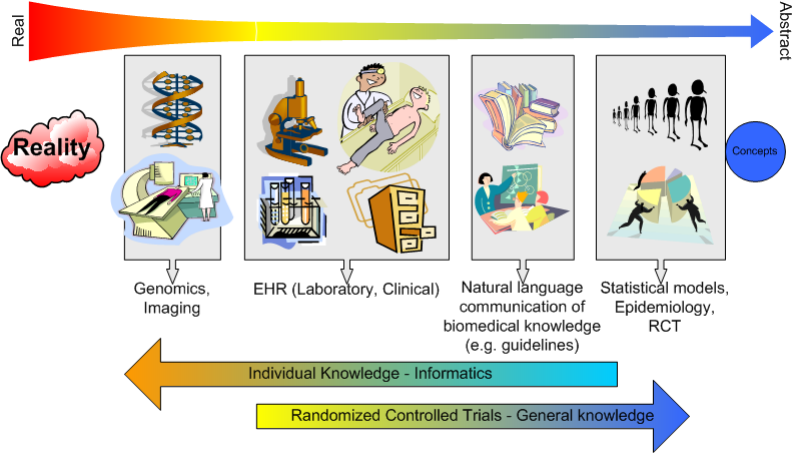
Biomedical knowledge on the knowledge spectrum.

From the perspective of the knowledge spectrum, the driving forces of Health Informatics and RCT methodology seem to have opposite directions: while Informatics aims towards *individual knowledge *and personalized health care, the general knowledge gained through populational studies (e.g., RCTs) targets the ideal of universal applicability (Figure 6). The value of a single bit of data (e.g., a Yes/No answer to a specific question such as a past appendectomy) can be very relevant in a decision-making context if it reduces the overall uncertainty of knowledge. However, such individual bits of data are inevitably lost during the creation of general knowledge.

*Rigorously and expensively collected, general, populational level knowledge is useful only in situations where individual knowledge lacks (e.g., new drugs), providing the decision makers have access to it and are able to apply it to specific situations. However, general knowledge is unlikely to be used as such in many naturalistic decision-making processes, because it does not support the way expert decision makers think. The knowledge gap and inherent instantiation uncertainty manifested in the application of general knowledge does not fully enable the education of providers and patients which would require additional knowledge about individual contexts of successful or unsuccessful application instances. Informatics, on the other hand, by advancing individual knowledge processing, provides an alternative solution to the problems that arise from the use of general knowledge that targets universal applicability. An integral part of individual knowledge, genomic data is already recognized *[39,40]*as being of extreme importance for a solution to the problems of general knowledge*.

### Knowledge representation by formal methods

The application of formal knowledge representations to real problems suffers from a fundamental shortcoming: the frame problem. As explained above (see "The frame problem"), the frame problem can be recast as a problem of relevance. Given the capability of relatively effortless human relevance judgments, the frame problem seems a rather "artificial" creation, difficult to grasp and which usually goes unnoticed. In order to circumvent its abstract nature, Dennett uses a story-telling approach. However, the frame problem also applies to and could be illustrated from the perspective of humans, who in their first years of life, learn and can easily and efficiently reason about the side effects and the implicit changes of the complex four-dimensional spatio-temporal physical world in which they live. As this learning gradually becomes common sense knowledge, it causes us to efficiently determine the relevant implicit changes while ignoring the non-relevant ones for a given situation. For example, such facts as that the clothes we are wearing are moving with us while walking or traveling are most of the times irrelevant given the context of a planned trip. However, if the trip involves some rapid movement through the air such as riding a motorbike, suddenly wearing a sombrero becomes a relevant fact. As experts at managing our physical world, we are able, through an effortless but powerful mental simulation, to determine the relevance of such a particular fact. The recall of our personal experiences of moving fast through the air and of the dragging force of the air becomes paramount. *Therefore, intelligent agent must be endowed with efficient mechanisms for determining the relevance of particular facts for a decision*.

We suggest that what made the robots vulnerable was their creators' choice for knowledge representation and reasoning: the robots did not have quick access to implicit knowledge about the relevance of particular facts (i.e., records of problem solving instances) but only to explicit facts in frames which had to be employed in time-consuming, immense number of explicit relevance judgments about the effects of particular actions. Although they were supposed to be experts at their task, the robots were behaving like novices. The frame problem is not a problem of the knowledge representation per se, but a problem of the choice for representation of knowledge needed to solve time-constrained decisions. In other words, formal representations and logic reasoning work, but not in time constrained, complex situations.

**Figure 7:**
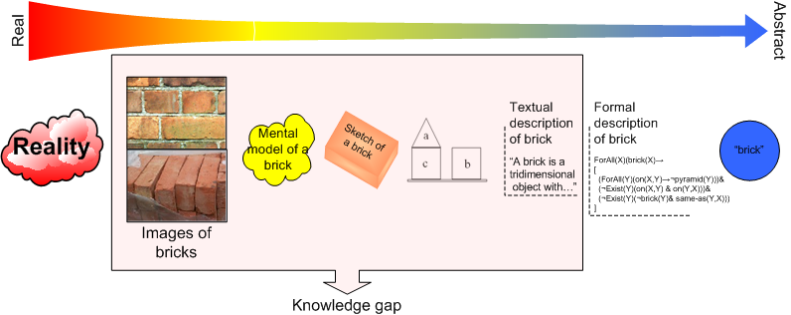
**Representations of "brick" on the knowledge spectrum. **Such representations range from rich (e.g., images, mental models) to less complex (sketches and diagrams) and to symbolic descriptions (textual, formal and conceptual).

From the perspective of our knowledge spectrum, explicit, formal representations sit on the abstract side of the spectrum (Figure 7). The retrieval of explicit knowledge representation is currently the subject of the increasingly important field of research of information retrieval (IR). It is commonly accepted that IR is strongly coupled with the notion of *intended meaning *of concepts: a retrieved document is considered to be relevant to a query if the intended meanings of the authors of a document are relevant to the intended meaning of that query. We propose that "meaning," a property that characterizes all concepts present in explicit knowledge, is intimately connected (if not identical) with the *notion of context*. According to this rather paradoxical view, meaning, a property which characterizes the abstract side of the knowledge spectrum, is strongly coupled with context which, by definition, is a feature of the reality side of the knowledge spectrum. Therefore, in order to construe meaning appropriately one needs to be able to efficiently move from abstractions towards richer representations of reality. This movement on the knowledge spectrum is necessary in order to fill the knowledge gap between abstract concepts and the richer mental representations required for construing their meaning.

Explicit, formal representations attempt to capture general truth and generally applicable problem solving strategies, but become too abstract in nature. Through the abstraction process, which is essentially a reduction driven by the relevance judgments of knowledge creators, the context of a problem is lost. Losing context creates difficulties with construing meaning (which is context-dependent by definition) and widens the knowledge gap between the representation itself and the reality of a future problem-solving instance. The knowledge gap translates into the instantiation uncertainty that characterizes the application of general knowledge to specific problems. Making up for the knowledge gap through explicit relevance reasoning becomes time consuming and consequently takes its toll on the applicability of the representation. In sensitive applications such as medical decision-making and health research, general knowledge may potentially be harmful (e.g., prescribing an highly recommended drug to which a patient has a undocumented allergy). In addition, abstractions and general methods and theories of problem solving and decision making (e.g., guidelines) do not fully enable the education of individuals and the learning from successes and mistakes.

*Knowledge representation approaches must therefore preserve to the extent possible, the context of a problem-solving instance. By efficiently recalling similar past instances of problem solving and their contexts, intelligent agents are immediately provided with implicit knowledge about relevance, encoded in the retrieved contexts and, in the same time, with more possibilities to reduce the instantiation uncertainty of general knowledge when applied to specific problems. To enable this, informatics research must advance the processing of rich representations of the knowledge encoded in past problem solving cases: this is the definition of CBR research*.

### Knowledge representation by natural language

Similar to formal specifications (e.g., predicate calculus) natural language uses abstractions, i.e., concepts. Its richness and power of expression place it in the knowledge spectrum to the left side of formal specifications but to the right side of rich descriptions consisting of images, sounds, video-clips and simulations of reality (Figure 4). Natural language has power of expression but loose semantics and inherent ambiguity. However, despite its abstract nature, it remains the indispensable, main knowledge representation and transfer medium between humans.

In order to illustrate our point about ambiguity we direct the reader to the previous, natural language definition of the concept of "a brick." Although the definition may look unequivocal, there are subtle ambiguities that make a difference in the predicate calculus representation. The first condition of an object to be "a brick" (i.e., "the brick is on something that is not a pyramid," highlighted in the equations 2 and 3) is an ambiguous natural language construction and could have slightly different formal representations:


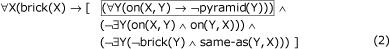



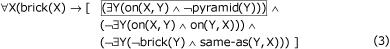


In (2) this condition has been interpreted as: "the brick being on something IMPLIES that that something is not a pyramid" and was therefore represented as "for all Y, if X is on Y, this implies that Y is not a pyramid." In (3), which is identical to (1) but is repeated to the benefit of the reader, this condition was interpreted as "the brick MUST BE (or is always) on something that is not a pyramid" and that was represented as "there exists Y such as X is on Y and Y is not a pyramid."

**Figure 8:**
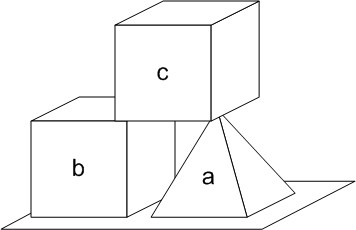
**A blocks world example. **In this particular example brick(b), brick(c), pyramid(a), on(c, b), on(c, a) are true and therefore not rejected by definition (3): the condition that "c" MUST sit on something that is not a pyramid in order to be a brick is met by on(c, b).

The first definition is therefore more "relaxed" as it allows the possibility that a brick sits on nothing. The second definition is more restrictive, because it requires the brick to be on something that is "not a pyramid" or otherwise X is not a brick anymore. Therefore, the first definition is more general and defines the concept of "a brick" in such a way that the definition would be true even in a world with no gravity (i.e., the brick is on nothing). In addition, definition (3) does not reject the possibility that an object sits on both another brick and a pyramid, at the same time (Figure 8).

The point is that, most often, humans receive and transmit knowledge without the deep understanding and completeness required by an exact mathematical representation of the knowledge to be transmitted. This shallowness has also been recognized by others [42] who are trying to draw natural language processing researchers' attention to the fact that humans are rather superficial in their knowledge acquisition and processing and often make use of "underspecified" representations. Although, since the early days of science, scientists have fallen in love with the pure reasoning approaches, as they were reproducible, unambiguous means to express new knowledge, the problems with the use of classical predicate calculus as a knowledge representation method and of the classical logic inference as a reasoning strategy are discouraging. This is due to the requirements of complete, unequivocal representations, which prevents them from dealing with the messiness of the real world problems.

If possessing the necessary knowledge, humans are able to effortlessly fill the knowledge gaps between natural language representations and their richer representations of reality (i.e., mental models), and to easily construe the appropriate meaning of potentially ambiguous concepts. Although current technology allows for its storage, knowledge present in richer media (e.g., images, videos, simulations) is currently very difficult to process (e.g., real-time computer vision, scene understanding and synthesis, image understanding) using today's technology.

*Because natural languages are used by people universally and allow rich representations that no other language specification can attain, natural language processing (NLP) research is a first step that Informatics should take in order to advance the organization and processing of individual knowledge in case-bases that can be reused. The insights gained will advance knowledge processing towards richer knowledge representation media, will reduce the knowledge processing gap and consequently increase the knowledge processing capacity currently supported largely by human knowledge processors*.

### Memory-based knowledge processing

One of the main features of information processing systems is their memory. It is accepted that storage and manipulation of information are necessary for complex cognitive activities in humans [43]. Memory is also considered crucial for both the "situation recognition" and mental modeling processes which are part of naturalistic decision models [33].

From a computational point of view, one could easily argue that without a random access memory structure there can be no effective processing. In the context of "the computational architecture of creativity," this argument is clearly outlined in [44]. It is based on the examination of the classes of computational devices, in the ascending order of their computational power, ranging from finite-state machines to pushdown automata and linear automata. These are paralleled by their corresponding grammars, arranged similarly in the Chomsky hierarchy, consisting of regular grammars, context-free grammars, context-sensitive grammars and of the unrestricted transformational grammars for machines with random access memory [44].

Recent natural language processing (NLP) research stresses the importance of memorization of individual natural language examples [45]. The importance of memory is also emphasized in earlier [46] and more recent models of language processing in humans [47-49]. These converge on the idea that natural language processing, regardless of the processor, is memory-based (i.e., case-based). Additional evidence comes from the fact that most language constructs (e.g. words, phrases) have very low frequencies. In fact, the very low frequency of most words in the English language (i.e., Zipf's law) is known from the 1940s since Zipf's famous book "Human Behavior and the Principle of Least Effort" [50] which is discussed in [51]. The main implication of "Zipf's law" is that purely statistical approaches or language processing algorithms that do not memorize training examples will either lose important information or may need extensive data (potentially impossible to collect) in order to be able to retain important features which have extremely low frequencies [52] and which may be crucial for construing the appropriate meanings of a language's concepts.

The tradeoff between learning effort and communication efficiency seems to be biased naturally towards memorization rather than towards logical reasoning. The processing complexity of natural language might therefore not be an intrinsic quality of the algorithms, but rather a function of the memorization capabilities of the language processor, given the sparseness of natural language pattern space. By analogy, the advanced knowledge processing in humans might not be the result of very sophisticated reasoning strategies, but rather the utilization of a limited reasoning apparatus on a huge knowledge base, consisting of rich representations of one's experience. The limitations in reasoning are balanced by complex spatio-temporal pattern recognition capabilities operating on a case base built from years of experience. This case base includes common-sense knowledge.

Furthermore, people and computers memorize information differently. Both have a short term, working memory and long-term memory for storing data and information. However, the memory access is carried out in different ways. Computers can reliably store large streams of data, which most of the times have a very well defined spatial and temporal structure (e.g., a movie clip). In contrast, people can only store information and knowledge rather than data and their storage is unreliable, temporally fragmented and spatially incomplete. Computers have very reliable memories capable of error checking at the bit level while the human memory supports only a high-level semantic consistency check. Finally, computers access their memory in a random seek fashion, being able to position their "reading heads" at any position in the data streams in order to extract a certain block of data. People can access their memory by content, by being provided with an incomplete description of a potentially complex, spatio-temporal pattern serving as a retrieval key. Therefore, one of the main differences between computers and humans is that computers have address-based random access memories, while humans possess content-addressable memories.

*In conclusion, from a case-based reasoning perspective, humans seem to be naturally endowed with the necessary structures for efficient case base acquisition, organization and retrieval while computers do not directly support this way of processing information and knowledge*.

### Pattern recognition, comparison and analogy-making

Pattern recognition is an undisputed feature of human cognitive abilities and a research area in its own right. However, it does not seem to be as pervasive as it should, in the information processing systems in current use. Natural language, as a product of human cognition, offers compelling evidence that people are naturally inclined toward processing information using pattern recognition and similarity principles. This evidence is supported by the widespread use of language devices such as the simile and the metaphor. These are examples of comparison and analogy making that humans perform without effort, in contrast to the difficulty of implementing them in the artificial information processing systems [53]. Analogy making is essential to generating new knowledge and new artifact designs [54-56], as well as to problem solving and inductive reasoning [57,58]. In a case-based reasoning context, the essential tasks of case matching and retrieval rely on pattern recognition, comparison and analogy making. In a decision making process, these mechanisms provide the immediate, implicit access to information about relevance stored in the contexts of similar instances of problems solving.

The patterns and analogies that humans are able to handle are often represented by complex spatio-temporal events with a potentially multi-sensorial impact. For example, while humans have no difficulty in understanding a metaphor like "the computer swallowed the disk," an artificial information processing system that has no visual input sensors and which lacks the capability of image understanding, would probably never be able to perceive this particular analogy with the same speed, because of the extensive reasoning and amount of explicit knowledge needed to bring the swallowing process, as it occurs in living things, close to the action of inserting a disk into a computer's disk drive.

In addition to operating on high dimensional, spatio-temporal complex patterns, analogy making in humans may also possess a dynamic component that could yield different relevance judgment outcomes, depending of context. A very illustrative example is given by French and Labiouse in [59], using the concept of a "claw hammer." According to its designed purpose, the "claw hammer" is semantically close to other concepts like "nail," "hit" and "pound." However, it may be dynamically "relocated" or reassigned in the semantic space, through a complex spatio-temporal mental simulation and analogy-making process, to the dynamically created class of "back-scratching devices," in the semantic neighborhood of the "itch," "scratch" and "claw" concepts. Similarly, one could think about the concept of a wooden decoy duck, which inherits properties from at least the "wooden object", "animal duck", "toy" and "hunting gear" classes. This concept may also be dynamically relocated into the semantic neighborhood of any of the classes, depending on the context of use that may be focused on themes such as "combustibles" or "hunting" for example. In the medical domain, the contextual dependence of relevance judgments, classifications and analogies is even more important, as these are often based on uncertain information and may be dynamically reevaluated in the light of new information about the patients or about their diseases.

Polyhierarchy and multiple inheritance are indisputable desiderata of terminology systems [60]. However, building multiple inheritance mechanism using current technology seems very difficult, simply because the number of possible alternative classifications increases exponentially with the number of concepts. It is also very unlikely that this kind of taxonomic dynamicity (e.g., the claw hammer circumstantially classified as a back-scratching-device) of the human semantic space could work on such fixed conceptual structures which are constructed beforehand through learning, in human semantic memory. A more plausible hypothesis is that such ad-hoc classifications are circumstantially created using mechanisms that are closer to a distance calculation between high dimensional, distributed, vector representation of concepts. This is in agreement with neurolinguistic evidence from functional brain imaging studies of the human semantic memory. These studies suggest the existence of distributed feature networks for the representation of object concepts [61] and help the case for less structured approaches to capturing and representing semantics such as compositional terminology schemes (e.g. as in GALEN-GRAIL [62] and SNOMED-RT [63]), latent semantic indexing (LSA) [64-70] and connectionist models [49,71,72]. These approaches allow for a multidimensional semantic space where concept features can vary in importance, evolve or change dynamically, accounting for many possible classifications and subtle variations of concept meaning, including the new and the less plausible ones. This contrasts with the fixed or highly structured semantic representation schemas (e.g. fixed knowledge frames, semantic networks, ontologies), which fail to capture concept semantics in a way that provides richness, dynamicity and reusability.

The dynamicity of concept meanings and relevance judgments may offer at least one of the reasons why fixed classification schemes, controlled terminology systems or open domain ontologies have not turned out satisfactory. It may also explain why existing lexical databases based on carefully handcrafted knowledge such as WordNet [73] often contain either too fine-grained or too coarse-grained, "static" semantic information [64]. In information intensive domains like medicine, concept dynamicity may account for why the development of a universal (i.e., one size fits all) clinical terminology system is so difficult [74].

*From a case-based reasoning perspective, humans are naturally equipped with powerful pattern matching and classification capabilities which allow them to cope with complex, time-constrained relevance judgments, to easily construe meaning of concepts and to tolerate the ambiguity of natural language*. Only relatively recently have computers come close to this functionality with the introduction of data mining and machine learning techniques such as self organizing maps and clustering algorithms based on similarity metrics [75]. In such machine learning approaches, the important problem of *feature selection *equates to a problems of relevance.

### CBR enabled EHR – Proposals, Challenges and Solutions

Iatrogenic causes are said to be important causes of death in the US [76]. The reported incidence of adverse effects among patients in Canadian acute care hospitals is 7.5% [77]. A proposed means to counteract such medical errors is information technology, through the education and decision support offered to health care professionals.

One very effective form of medical education is the retrospective analysis of case records where health professionals, both experienced or novices, learn from their own and from others' successes and failures [78]. Providing that legal and ethical implications such as provider and patient protection are dealt with appropriately, the efficacy of this teaching method can be improved if case records are continuously created, enriched, accumulated and organized on similarity principles. This is possible through a CBR approach of the EHR which, from this perspective, could serve as a comprehensive case base of managed patients that will evolve asymptotically towards an exhaustive knowledge base.

Medical errors are also connected with the complex human cognitive task of planning [79]. CBR approaches, devised originally as a solution to automated planning tasks [80], have been since used in various applications including healthcare, legal and military (e.g., battle planning) [21]. This demonstrates a particularly good fit of a medical decision support based on CBR with its human users, the healthcare professionals.

Providing that the privacy and confidentiality issues, which are even more stringent in this case, are dealt with appropriately, opening EHR to patients could benefit them [81]. It is perfectly conceivable that patients could learn from the history of other cases similar to theirs, which could be presented in an anonymized, story-telling format and organized on case similarity principles. It is also possible that patients may be willing to directly provide some of their own case information in order to be matched with previously managed cases, for example in the context of online chronic disease support groups. These principles are already realized in form of bulletin boards, mailing lists and forums, where actual patients interact with each other and occasionally with health professionals and exchange information regarding health related problems ([82] and [83]). The unstructured, textual exchange of information in such resources would ideally be moderated by knowledgeable individuals (e.g., providers). Although the automatic processing of text still is not readily available, case matching is possible so far and is performed by the very individuals who are able to offer useful information and knowledge to others, based on the similarity of their own experiences (i.e., their own story).

Medicine has always and will always be a case oriented profession. Medical Informatics has recognized this early through the works of various researchers who pioneered the area of decision support systems [84]. Relevant to CBR work are also the attempts to enhance early decision support systems with domain knowledge from simulated patient cases [85]. Currently, the exploration of CBR in medical contexts is increasing [86-94]. Regardless of the problem nature, the most important components of a CBR expert system are

• The case base, the memory of past problem-solving instances

• The case matching or pattern matching procedure which retrieves the relevant cases for a certain problem

While humans seem to possess a natural support for these two components, there is still work to be done in order to make the computer support this kind of knowledge acquisition and processing. We envision four important challenges in advancing towards CBR enabled EHRs:

1. Case record comprehensiveness

2. Organization on similarity and associative principles (associative memory) and development of advance data visualization techniques

3. Development of pattern recognition and similarity measures between heterogeneous records

4. Solving ethical issues and provision of privacy and confidentiality measures

#### 1) Case record comprehensiveness

EHR comprehensiveness is required because the exhaustiveness of a case base is not only a function of the number of records but also of the richness of each case record. Current knowledge processing technology limits the acquisition and especially the processing of comprehensive EHR records which incorporate structured data, images, video-clips, bio-signals, genomic data, unstructured textual data covering clinical findings, detailed patient history, etc. However, as knowledge processing technology advances and knowledge acquisition bottlenecks are overcome, it might be possible to overcome the heterogeneity and sparseness of EHR and allow the creation of representative case-bases and the organization of knowledge on principles that facilitate similarity based retrieval.

Temporal knowledge is also a good example of a heterogeneously represented type of knowledge in the form of potentially non-interoperable standards for dates and times and temporal knowledge of various degrees of precision, embedded in knowledge facts such as "soon after receiving the drug, the patient developed a rash." Currently, for many people, the problem may seems to boil down to devising yet another standard which encompasses all the different temporal representations of dates, times and temporal concepts into a unified, common representation. From a knowledge engineering standpoint, and again currently for many researchers, this may equate to the creation of a comprehensive ontology of temporal knowledge. However, the problem of representing time starts to look like a somewhat limited version of another burning problem of Medical Informatics: that of medical terminologies. The fact that all these issues remain largely unsolved, can only help the case for CBR and for adaptive, empirical methods and approaches to knowledge processing. We believe that such approaches have the potential to cope and overcome the problems with redundant, possibly ambiguous representations, which have arbitrary degrees of precision. Thereby we are specifying a goal towards which the development of EHR should proceed.

#### 2) Organization on similarity and associative principles (associative memory) and development of advanced data visualization techniques

**Figure 9:**
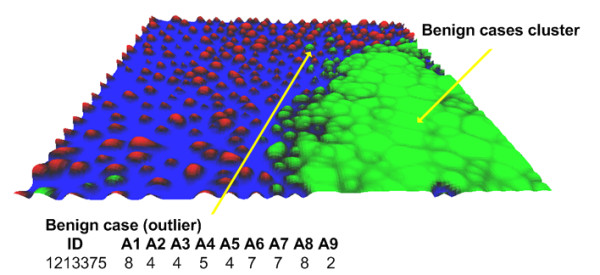
**Example of self organizing map (associative memory). **Each one of the 9-dimensional, 683 individual cases is associated with a region on the 2-dimensional map and highlighted using a 3rd dimension (an "activation bubble" with elevation and colour). The organization of individual descriptions of cases obeys similarity principles: similar cases are closely mapped and very similar cases form clusters (e.g., the green area contains most of the benign cases).

Similarity based retrieval is difficult with current database technology. For example, queries to retrieve cases which are similar to a textual description of a given case are difficult to answer. The comprehensiveness of EHR must be complemented with the possibility of indexing its records on similarity principles. Conceptually, the functionality of EHR will be that of an associative memory of cases that will enable the CBR paradigm. The organization of a case-base must be complemented by the development of advanced data visualization techniques that comply with the principles of organization of information by similarity. One example of such data visualization techniques are self-organizing maps [75]. These models are able to perform cluster analyses on high dimensional data sets and provide a visual display which can help with the navigation through and retrieval of similar cases. For instance, the self-organizing map obtained from the analysis of the Wisconsin Breast Cancer Dataset [95] used to cluster and classify cases based on their similarity in [96], could also be used for data visualization and navigation purposes, in a CBR context (Figure 9). It also demonstrates how high level abstractions (i.e., benign tumors forming the green cluster on the map) can be derived through an entirely automatic, data driven approach.

#### 3) Development of pattern recognition and similarity measures between heterogeneous records

CBR relies on the proper management of the case base and on appropriate mechanisms for matching and retrieval of these case records. All similarity retrieval mechanisms are based on some sort of distance calculation between the problem at hand and the records in the case base, followed by the retrieval of the most relevant ones. Clinical narratives and other EHR components containing unrestricted text represent a particularly difficult challenge for semantic similarity measures. The development of terminology systems based on less structured (e.g., latent semantic indexing, connectionist models) and data-driven approaches will provide the semantic richness, dynamicity and reusability needed for such complex tasks.

**Figure 10:**
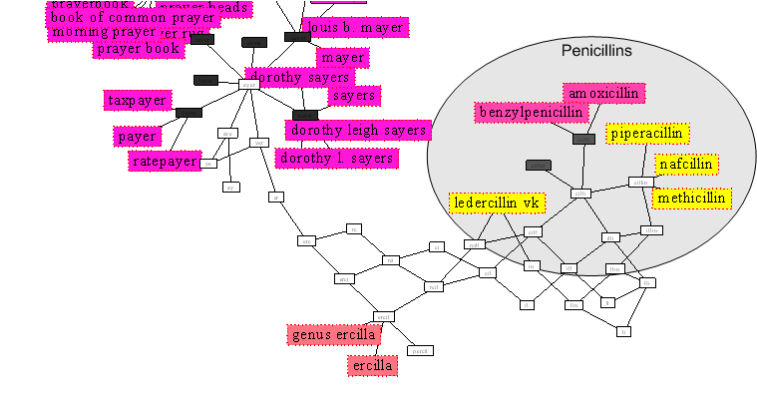
**Example of similarity based retrieval and knowledge induction. ***Ayercillin*, a relatively new drug unknown to the system, is likely to be a penicillin because of its high contextual similarities with known penicillines.

A concrete example for the potential feasibility of such approaches, is the automated knowledge induction based on contextual similarity modeling ranging from morphological to sentential context [97] (Figure 10). An experimental knowledge processing system can induce automatically the new knowledge fact that *Ayercillin*, an item unknown to the system and hence not appearing in Figure 10, is most likely to be a drug, precisely a penicillin. The decision is based on morphological (e.g., "-cillin"), semantic (e.g., six of the similar items are known to be drugs, precisely, penicillins) and pragmatic (e.g., the six, semantically similar items are consistent with the use in a medical context) similarities that help in filtering out the non-relevant information (e.g., book of common prayer). On the same basis, the system can also induce that surgical procedures ending in "-tomy" (e.g., perineotomy, valvulotomy, myringotomy, strabotomy) are usually incisions while those ending in "-ectomy" (e.g., myringectomy, tonsillectomy, splenectomy, nephrectomy) are usually removals, that concepts containing the morpheme "leuco" (e.g., leucocyte, leucothoe, platalea leucorodia) are usually associated with color white while those containing "eryth" (e.g., erythroblast, erythema, erythrina) with color red.

However, despite such proof-of-concept applications and other progress in data mining and knowledge extraction from heterogeneous databases, case matching remains largely an open research question.

#### 4) Solving ethical issues, provision of privacy and confidentiality measures

We discuss this challenge last, not because it is less important but, on the contrary, because of its potential to become the most important obstacle to individual knowledge processing. The very fact that individual knowledge has the potential to contribute to solving future problems instances, raises the important ethical issue whether such knowledge should be made available to decision makers and researchers. Because the definition of individual knowledge implies the possibility to match it in time and space with an application context, i.e., with a patient, sharing individual knowledge is counterbalanced by the need for privacy and confidentiality. In addition, to further complicate matters, it may turn out that some of the most useful records for future instances of decision making are instances of medical errors or other unexpected events that are unique in their course of events and therefore easily identifiable together with their contexts of development (i.e., patients, providers, family members).

The high complexity of individual knowledge renders explicit, manually controlled access to individual knowledge cases and their components unfeasible. The only solution to this problem seems to be of technological nature. Current privacy and confidentiality measures which include de-identification, de-nominalization and scrambling of the unique personal identifiers automated or semi-automated seem insufficient to counteract the potential to identify patients from unique, individual knowledge patterns.

As a general approach, we propose that the accurate measuring of similarity of individual knowledge could form the basis of a confidentiality risk assessment. This could be intuitively understood by considering that:

• very similar individual knowledge patterns which are in great numbers are a very low threat to the privacy and confidentiality infringement, and, at the other extreme,

• stand-alone patterns which possess unique features or combinations of features, are at high risk of privacy and confidentiality breaches.

In addition, the provision of privacy and confidentiality could be regarded as a special case of knowledge processing, which involves *knowledge about the proper use (e.g., access, modification, transfer) of individual knowledge*. This potentially complex, particular case of meta-knowledge processing could be implemented and managed using the principles of CBR paradigm itself, by building case-bases with examples of both proper and improper (simulated, not necessarily real) individual knowledge accesses and that can be compared with future access instances.

Overcoming this very important challenge hinges on the possibility to effectively measure the similarity between heterogeneous records and on the advancement of knowledge processing on CBR principles.

If successful, CBR research might therefore fulfill a longstanding need for intelligent information processing and advance informatics towards the ideal of individual knowledge processing. This calls for further investigation of information processing models that are, similarly to human experts, capable to efficiently move across the knowledge spectrum. One class of such models is represented by artificial neural networks [14,23], which are highly adaptive information processing models able to create high-level abstractions from raw data, completely automatically [75] and "learn by themselves" new information processing functions from data. From this perspective, Informatics aligns closely to the goals of AI to create intelligent machines that can hear, see, speak, think, adapt and make decisions.

## Conclusions

CBR provides potential solutions to important problems that, among other, stymy the usefulness of EHR. The natural integration of learning with reasoning and the CBR resemblance to the cognitive models of human decision-making hold the promise to overcome the "brittleness" and "knowledge acquisition bottleneck" of classical expert systems. The CBR applications to the medical field have the potential to offering the training and decision support needed by health professionals and the means towards a true patient-centered healthcare.

With a CBR theoretical foundation still in its infancy and with limited medical applications in existence, more research is needed for providing proofs of the feasibility of practical CBR-EHR applications. Challenges in the way to accomplish this include the increasing complexity, ethical issues as well as the paradigm shift that our current computing devices must undergo in order to support the CBR principles of knowledge processing.

## Summary

1. Science is twofold and is driven by two opposite forces: that of creating theories (theoretical sciences), and that of applying theories to practical applications (applied sciences). Medical Informatics is fundamentally an applied science that should be committed to advancing patient-centred medicine through individual knowledge processing.

2. Case-based reasoning is the technical solution that enables a continuous individual knowledge processing that could be integrated with the Electronic Health Records.

3. Medicine is an information and knowledge intensive domain where time-constrained decision problems can only be solved effectively based on the recollection of similar problems and their solutions (i.e., a case-based reasoning strategy). The collective sharing of experiences is important for making future decisions as well as for learning how to make decisions.

4. Unlike computers, human decision makers possess the components necessary to perform case-based reasoning naturally (i.e., a content addressable memory to organize a case base efficiently by similarity principles, as well as the capability to perform pattern recognition, comparison, and analogy-making).

5. Applying the CBR approach to EHR might be a way to overcome the important obstacles of EHR acceptance and use, providing that technical challenges and ethical issues arising are addressed appropriately.

## List of abbreviations

AI Artificial intelligence

AIT Algorithmic Information Theory

CBR Case Based Reasoning

E Explicit knowledge

EHR Electronic Health Records

G General knowledge

LSA Latent Semantic Indexing

I Individual knowledge

NDM Naturalistic Decision Making

RCT Randomized Controlled Trial

RPD Recognition-Primed Decision

U Implicit (Unobvious) knowledge

## Competing interests

The author(s) declare that they have no competing interests.

## Authors' contributions

### Before the reviews

SP researched the paper and provided a first draft. JA, JM critically revised the manuscript three times each and provided their own additions to the initial draft. JA provided more feedback on the cognitive aspects and decision-making as well as writing style and missing references. JM additions were with regard to the writing style, clarity, missing references and the overall organization of the paper.

### After the reviews

SP and JM worked on the responses to reviewers' comments. SP wrote a first revision of the paper. JM provided extensive feedback as well as new references and suggested a major revision that includes recent ideas. JA also commented and made suggestions on the knowledge spectrum model and on the meta-level view on Medical Informatics. SP overhauled the entire paper. JM revised the new version and provided feedback. SP operated the changes and proposed new modifications. JM revised the second draft. JA provided feedback on the second draft of the paper with regard to fundamental aspects of knowledge modalities.

SP and JM incorporated the minor changes suggested by the last review.

All authors read and approved the final version of the paper.

## Pre-publication history

The pre-publication history for this paper can be accessed here:



## Figures and Tables

**Table 1:** Implicit and explicit knowledge

	**Implicit knowledge (U)**	**Explicit knowledge (E)**
**Example**	The implicit knowledge used to recognize the face of a specific person.	The explicit knowledge (e.g., textual descriptions) that would allow to recognize faces of people (including a specific person).
**Complexity, Context retention**	Rich, grounded in reality. High retention of context in form of salient features.	Lean, more abstract, symbolic. Variable amount of context retention.
**Acquisition**	Detection, learning of correlations and regularities of environment.	Explicitation of one's implicit knowledge. Explicit acquisition of knowledge (e.g., through reading).
**Representation**	Unstructured, present implicitly in data recordings of the environment (e.g., image of a person).	Varies from less structured (e.g., natural language) to very structured (e.g., formal descriptions).
**Transferability**	Transferable only in implicit form through the data recordings (i.e., representations) of the environment.	Transferable through languages (natural or formal) and communication (e.g., verbal).
**Applicability**	Very well applicable to specific problem instances.	Applicable to both, specific and more generic problems.
**Processing mechanisms**	Pattern recognition, feature selection, associative memory.	Reasoning.

**Table 2:** Individual and general knowledge

	**General knowledge**	**Individual knowledge**
**Example**	Explicit general propositions, rules, algorithms, guidelines and formal theories for recognizing faces of people (e.g., a formal theory of human face recognition).	The implicit knowledge used to recognize and the explicit knowledge (e.g., textual description) that would allow recognizing the face of a specific person.
**Complexity**	Very lean, abstract, symbolic.	Varies from rich to lean.
**Acquisition**	Identical to acquisition of explicit knowledge.	Identical to acquisition of both implicit and explicit knowledge.
**Representation Transferability**	Very structured, highly transferable, explicitly as general propositions, rules and guidelines.	Varies from unstructured to less structured. Transferable in both implicit and explicit form.
**Context retention Applicability**	Does not retain context. Easy applicable to generic problems, difficult to apply to specific problem instances (e.g., recognition of the face of a specific person).	Retains context. Well applicable to specific problem instances, especially if context retention is high.
**Processing mechanisms**	Logic reasoning.	Pattern recognition, feature selection, associative recall, case-based reasoning.
